# Editorial: Systems Biology and Synthetic Biology in Relation to Drought Tolerance or Avoidance in Plants

**DOI:** 10.3389/fpls.2020.00394

**Published:** 2020-04-09

**Authors:** Xiaohan Yang, John C. Cushman, Anne M. Borland, Qingchang Liu

**Affiliations:** ^1^Biosciences Division, Oak Ridge National Laboratory, Oak Ridge, TN, United States; ^2^Oak Ridge National Laboratory, The Center for Bioenergy Innovation, Oak Ridge, TN, United States; ^3^Department of Biochemistry and Molecular Biology, University of Nevada, Reno, NV, United States; ^4^School of Natural and Environmental Science, Newcastle University, Newcastle upon Tyne, United Kingdom; ^5^Key Laboratory of Sweet Potato Biology and Biotechnology, Ministry of Agriculture, Beijing Key Laboratory of Crop Genetic Improvement, Laboratory of Crop Heterosis and Utilization, Ministry of Education, College of Agronomy & Biotechnology, China Agricultural University, Beijing, China

**Keywords:** genomics, synthetic biology, systems biology, drought tolerance, drought avoidance, crassulacean acid metabolism

Drought stress has been a long-time limitation to crop production that is being exacerbated by climate change and associated reductions in the availability of blue water resources for agriculture. Most existing food and industrial crops are susceptible to drought stress, which can cause a significant loss in crop yield. Therefore, our ability to develop more climate-resilient crops that are more heat and drought tolerant will become increasingly important in the near future. In nature, plants have evolved two important mechanisms to overcome the effects of drought stress: (1) drought avoidance, which enables plants to maintain relatively high tissue water content in water-limited environments by minimizing water loss and optimizing water uptake, and (2) drought tolerance, which enables plants to endure low tissue water content by maintaining cell turgor (resulting from osmotic adjustment and cellular elasticity) and increasing protoplasmic resistance (Basu et al., 2016). With more and more genomics resources available for diverse plant lineages showing contrasting strategies and variation in drought avoidance or tolerance (Yin et al., 2014; Abraham et al., 2016; Yang et al., 2017; Chen et al., 2018), systems biology, which features genome-scale analysis of molecules and their interactions (Westerhoff and Palsson, 2004), is becoming a popular approach to link genes to drought-avoidance or drought-tolerance traits. Our knowledge about the genes associated with drought stress responses generated by systems biology research can inform the construction of libraries of biological parts for synthetic biology, which aims to design or re-design biological processes (Cook et al., 2014). Synthetic biology has great potential for creating genetically-modified plants with enhanced drought avoidance or tolerance (Borland et al., 2014; De Paoli et al., 2014; Llorente et al., 2018). This Research Topic features three articles on the theme of systems biology of crassulacean acid metabolism (CAM) as a model strategy for plant adaptation to water-limited conditions and four articles related to genetic improvement of plant drought avoidance or tolerance using synthetic biology and genetic engineering approaches.

## Identification of CAM-related Genes Using Systems Biology Approach

CAM photosynthesis enables drought avoidance and high water-use efficiency (WUE) in plants through an inverted day/night pattern of stomatal movement relative to C_3_ or C_4_ photosynthesis plants, with stomata closed during daytime to reduce water loss via evapotranspiration (Borland et al., 2009; Yang et al., 2015). In this Research Topic, Moseley, Tuskan et al., compared diel gene expression patterns between one obligate CAM species *Kalanchoë fedtschenkoi* and two C_3_ photosynthesis species (*Arabidopsis thaliana* and *Solanum lycopersicum*). They identified 16 ortholog groups (OGs) containing stomata-related genes showing rescheduled (dawn vs. dusk) gene expression in the CAM species in comparison with the two C_3_ species. Furthermore, they performed evolutionary genomics analysis of these 16 OGs and highlighted several genes, such as serine/threonine-protein kinase nak1 and Catalase 2, as candidates regulating stomatal movement in CAM plants *via* abscisic acid (ABA) signaling and hydrogen peroxide (H_2_O_2_) signaling, respectively. Also, in this Research Topic, Heyduk et al. performed comparative analysis of time-course gene expression between two closely-related species in the genus *Erycina, E. pusilla* and *E. crista-galli*, which are CAM and C_3_ photosynthesis plants, respectively. Their analysis revealed differential expression networks of genes involved in light sensing and ABA signaling between the C_3_ and CAM *Erycina* species. CAM physiology is likely controlled by the circadian clock (Boxall et al., 2017; Yang et al., 2017). In this Research Topic, Moseley, Mewalal et al. performed genome-wide prediction of rhythmic gene sets in the CAM species *K. fedtschenkoi* and C_3_ photosynthesis species *A. thaliana* through analysis of time-course gene expression data and identified CAM-related rhythmic genes, which displayed phase shifts between these two species. Recently, knock-down and knock-out mutant lines were generated for some CAM-related genes using RNA interference (RNAi) (Boxall et al., 2017, 2020) or genome-editing mediated by clustered regularly interspaced short palindromic repeats (CRISPR) and CRISPR-associated protein 9 (Cas9) (Liu et al., 2019). Multi-scale modeling of CAM systems at the molecular, cellular, and leaf level is needed to gain a deep understanding of gene and metabolic networks associated with CAM physiology (Liu et al., 2018). In the future, multi-omics (i.e., genomics, transcriptomics, metabolomics, proteomics) data will need to be generated for CAM plant species and their mutant lines for constructing gene and metabolic networks relevant to CAM.

## Genetic Improvement of Plant Drought Resistance Using Synthetic Biology and Genetic Engineering Approaches

Engineering of CAM-related genes in C_3_ plants has great potential for genetic improvement of water-use efficiency and drought resistance (Borland et al., 2014; Yang et al., 2015). Comparative analysis of leaf metabolic networks between CAM and C_3_ plants indicates that CAM-engineering in C_3_ crops could result in a major increase in water-use efficiency without substantial negative impact on yield (Shameer et al., 2018). CAM-engineering is likely to prove a challenging task, requiring a synthetic biology approach for precise control of temporal and spatial expression of multiple gene modules responsible for carboxylation, decarboxylation, stomatal movement, and leaf anatomy. In this Research Topic, Lim et al. showed exciting progress in transferring individual CAM carboxylation and decarboxylation genes from the facultative CAM species *Mesembryanthemum crystallinum* (ice plant) to the C_3_ species *A. thaliana*. Over-expressing each individual gene of the *M. crystallinum* carboxylation module (containing 6 genes) and decarboxylation module (containing 7 genes), except for three genes (*McNADP-MDH1, McPPDK-RP*, and *McPEPCK*), under the control of the constitutive CaMV 35S promoter, increased plant size (in term of rosette diameter, leaf area, and leaf fresh weight) in the transgenic *A. thaliana* plants. Over-expression of most carboxylation genes increased stomatal conductance and acid accumulation while over-expression of the decarboxylating malic enzymes reduced stomatal conductance and acid accumulation. This study is an important milestone in CAM-engineering. Future research will be needed to test the coordinated over-expression of combinations of these carboxylation and decarboxylation genes in the same temporal and spatial manner as displayed by CAM species.

In general, drought stress experienced by crop plants is seasonal. Therefore, engineering drought-inducible CAM (or CAM-on-demand) systems would be ideal. In nature, CAM can be induced by drought stress in facultative CAM plants, which perform C_3_ photosynthesis under well-watered conditions (Winter, 2019; Yang et al., 2019a). In this Research Topic, Amin et al. proposed a strategy for engineering of CAM-on-demand systems based on the engineering of drought-responsive transcription factors (TFs) in multiple gene families (e.g., *AP2/ERF, MYB, WRKY, NAC, NF-Y, bZIP*) from the facultative CAM plant *M. crystallinum* and obligate CAM plant *K. fedtschenkoi*. The study showed that overexpression of a drought-responsive NAC family gene from *K. fedtschenkoi* (*KfNAC83*) in *A. thaliana* enhanced resistance to water-deficit stress and increased WUE. In the future, establishing the regulatory relationship between the drought-responsive TFs and CAM pathway genes will be necessary to establish CAM-on-demand systems.

Drought-responsive genes in C_3_ or C_4_ photosynthesis plant species are a rich source of target genes for genetic engineering to improve drought tolerance (Umezawa et al., 2006; Kamthan et al., 2016). In this Research Topic, Chen et al. increased the drought tolerance in rice through over-expression of a drought-inducible rice gene *OsNAR2.1* encoding a nitrate transporter partner protein. Also, in this Research Topic, Lian et al. demonstrated that ectopic expression of a *Populus trichocarpa* gene (*PtNF-YA9*), which encodes a NUCLEAR FACTOR Y transcription factor, enhanced drought tolerance in the vegetative growth stage in *Arabidopsis*. A key focus for future research will be to test if overexpression of *PtNF-YA9* can increase biomass production in poplar, an important C_3_ bioenergy crop, under drought conditions. Besides *OsNAR2.1* and *PtNF-YA9*, many other genes are also responsible for drought resistance (Singh et al., 2019). In the future, genetic circuits (e.g., toggle switches, feedback loops, Boolean logic gates) (Kassaw et al., 2018) combining CAM genes and drought responsive TFs through iterative design-build-test-learn cycles will be required for drought avoidance and drought tolerance, respectively. For example, Boolean logic gates, which utilize Boolean algebra to convert multiple input signals into truth values of 1 if true or 0 if false (Andres et al., 2019), can be used to control the expression of genes related to drought tolerance through integrating drought-induced positive and negative transcriptional regulators. Also, synthetic oscillator comprising positive and negative feedback loops (Andres et al., 2019) and circadian clock-regulated toggle switch (Schmal et al., 2013) need to be created for controlling the temporal (i.e., day vs. night) expression of genes related to CAM and stomatal movement in the C_3_ plant species.

## Concluding Remarks

This Research Topic has identified important candidate genes underpinning drought avoidance and tolerance through systems biology research and has also demonstrated the potential of synthetic biology and genetic engineering for increasing drought resistance in plants. With genomics research shifting from genome-reading to genome-editing and rewriting (Yang et al., 2019b), the knowledge generated in this Research Topic will facilitate future efforts in designing climate-resilient crops for reducing yield losses and expanding the production of food and bioenergy crops to marginal lands.

## Author Contributions

All authors listed have made a substantial, direct and intellectual contribution to the work, and approved it for publication.

### Conflict of Interest

The authors declare that the research was conducted in the absence of any commercial or financial relationships that could be construed as a potential conflict of interest.
